# Effect of Peri-Operative Blood Transfusion on Short and Long-Term Mortality Rates in Elderly Patients With Neck of Femur Fractures: A Retrospective Study

**DOI:** 10.7759/cureus.38825

**Published:** 2023-05-10

**Authors:** Vipul Garg, Vikram Kishor Kandhari, Omer Nasim, Yogesh Joshi

**Affiliations:** 1 Trauma and Orthopaedics, Wrexham Maelor Hospital, Wrexham, GBR; 2 Trauma and Orthopaedics, Glan Clwyd Hospital, North Wales, Chester, GBR; 3 Trauma and Orthopaedics, Poole General Hospital, Poole, GBR

**Keywords:** packed red blood cell transfusion, 30-day mortality, orthopaedics trauma, geriatric hip fracture, hip surgery

## Abstract

Introduction

The current reported mortality rate for elderly neck of femur fractures (eNOFF) is relatively high in the UK. eNOFF patients commonly suffer from associated cardiovascular co-morbidities and tend to have fragile physiological states and poor physiological reserves. Although some studies have shown a potential link between blood transfusion and mortality in eNOFF patients, there is no general consensus on this matter. Therefore, our study aims to explore the possible association between blood transfusion and length of hospital stay (LOHS) as well as short- and long-term mortality rates in eNOFF patients by reviewing the practice of blood transfusion.

Methods

This retrospective study was conducted at Wrexham Maelor Hospital, which is part of the Betsi Cadwaladr University Health Board (BCUHB), Wales. The study included patients who were 65 years of age or older and presented with neck of femur fractures. Only patients who required surgical intervention were included, and those managed non-operatively were excluded from the study. The statistical analysis was performed using IBM SPSS Statistics for Windows, Version 25.0 (IBM Corp., Armonk, New York, United States). Furthermore, unpaired t-tests and log-rank (Mantel-Cox) tests were performed to compare the groups that received blood transfusions.

Results

During the study period, a total of 501 eNOFF patients were included in the primary cohort of the study, with a mean age of 81 years (ranging from 65 to 102). The majority of the patients were female (n=340). Of the 501 patients, 79 (15.8%) received a blood transfusion during their treatment. Around 52.9% of the eNOFF patients were categorized as American Society of Anesthesiologists (ASA) III, but there was no statistically significant difference in the requirement of blood transfusion between patients in ASA III, II, and IV categories, as compared to ASA I. Additionally, the mean time to surgery was higher in patients who received a blood transfusion (35.8 hours), and this difference was statistically significant (p=0.035).

Moreover, the average LOHS after surgery for eNOFF was longer in patients who needed peri-operative blood transfusion (22 days), and this difference in the means was statistically significant (p=0.022). At the one-year post-surgery mark, mortality was higher in the transfused group (33%), and long-term five-year mortality rates were also higher in this group (63.2%).

Conclusion

Peri-operative blood transfusion may confer certain benefits in the management of eNOFF ptients. However, it should not be regarded as a panacea for improving long-term outcomes. The decision to administer blood transfusion must be made on a case-by-case basis, with careful assessment of individual clinical indications, and the potential risks and benefits taken into consideration. To achieve optimal clinical outcomes, close monitoring and follow-up of eNOFF patients, both in the short-term and long-term, are essential.

## Introduction

Elderly neck of femur fracture (eNOFF) is one of the most common orthopaedic injuries encountered worldwide by orthopaedic surgeons. The annual incidence of eNOFF in the United Kingdom (UK) is 70,000-75,000 [1]. Thus, it is imperative for any practising orthopaedic surgeon to be well acquainted with the peri-operative management of eNOFF patients.

The current reported 30-day mortality for the eNOFF is 8.3% in the UK [1]. eNOFF patients commonly suffer from associated cardiovascular co-morbidities and tend to have fragile physiological states and poor physiological reserves. They also tend to have a limited capacity for physiological autoregulation. An episode leading to eNOFF subjects the patient to significant physiological stress. On average, the haemoglobin level in these patients drops by 0.6-1.1 gm/dL, just because of the injury [2,3]. Most of these patients further undergo operative intervention for management. Further blood loss of about 3.1 gm/dL is expected secondary to the operative intervention in the postoperative period [4]. Thus, during an episode of eNOFF patients tend to further compromise their physiological status. If these patients suffer from low haemoglobin at the beginning of the episode, they will almost certainly require a blood transfusion in the pre or peri-operative period of their injury to optimize their level of haemoglobin. This is essential to cope with the physiological stress of injury and surgery. Also, moderately anaemic patients tend to struggle with rehabilitation and may have prolonged lengths of hospital stay (LOHS) [5]. Though blood transfusion is beneficial in such patients, it does not come without its risks.

There have been reports of increased morbidity and mortality secondary to increased rates of blood transfusion in eNOFF patients [6]. After a blood transfusion, the potassium and lactate levels increase in the recipient [7,8]. The change in the levels is usually small and is taken care of by the autoregulatory mechanisms. In eNOFF patients, these autoregulatory mechanisms are compromised and they may need multiple transfusions; thus, the effect of the biochemical changes can be profound and might contribute to increased mortality. Also, eNOFF patients commonly suffer from cardiovascular co-morbidities and may also have underlying chronic kidney disease. Cardiovascular complications have been a leading cause of mortality in eNOFF patients [9]. Increased blood volume secondary to blood transfusion may cause increased preload on the heart and may increase the risk of cardiac failure. Thus, blood transfusion may also be associated with increased mortality in eNOFF patients [6].

Though some studies have shown an association between blood transfusion and mortality in eNOFF patients, overall, there is a lack of consensus. Through this study, we aim to review the practice of blood transfusion and explore the possible association between blood transfusion and LOHS and short and long-term mortality in eNOFF patients.

## Materials and methods

This was a retrospective study conducted in the Wrexham Maelor Hospital located in Wrexham, Wales, a part of the Betsi Cadwaladr University Health Board (BCUHB). It included all consecutive patients admitted to the hospital under the care of the Department of Trauma and Orthopaedics who were diagnosed to have eNOFF between January 2019 and December 2021. The included patients were >/= 65 years of age presentation. The fracture patterns included were intracapsular, extracapsular, and subtrochanteric femur fractures. Only the patients who required surgical intervention (total hip replacement, hip hemiarthroplasty, dynamic hip screw fixation, intramedullary nail fixation, or percutaneous screw fixation) were included in the study. eNOFF patients who were managed non-operatively were excluded from the study. Patients who were diagnosed to have pathological eNOFF or who suffered eNOFF because of poly-trauma and suffered additional fractures were also excluded.

The demographic, medical history, operative, in-hospital treatment, and post-discharge details of the patients were accumulated from different online sources including the National Hip Fracture Database (NHFD), the transfusion lab information portal (Welsh Laboratory Information Management System (LIMS)), and the Welsh Clinical Portal. The information regarding the mortality of the patients was collected from the clinical information portal. We also used the actual patient notes from the hospital records section to add to any missing information. These resources are reliable clinical information portals for the patients treated under BCUHB. The requirement for blood transfusion was judged based on the patient's vital parameters, symptoms, and level of hemoglobin in the peri-operative period. This was also agreed upon by the treating orthogeriatrician in the ward-based multi-disciplinary team discussion.

Statistical analysis was performed using IBM SPSS Statistics for Windows, Version 25.0 (IBM Corp., Armonk, New York, United States). Mean, median, standard deviation (SD), maximum, and minimum values, as well as the 95% confidence interval (CI), were calculated for each variable. In addition, we used the Student unpaired t-test and log-rank (Mantel-Cox) test for independent samples to determine significant differences between the groups of patients who did and did not receive a blood transfusion. For all tests, P < .05 was considered significant.

## Results

Over the study period, a total of 501 eNOFF cases constituted the primary cohort of the study, with a mean age of 81 years (ranging from 65 to 102 years). Among the eNOFF cases, female patients (n=340) outnumbered male patients (n=161). Of the total cohort, 79 (15.8%) patients received a blood transfusion in the peri-operative period. While a higher proportion of female patients received peri-operative blood transfusions compared to male patients (61 out of 340 versus 18 out of 161), the difference was not statistically significant (p=0.07). Most of the transfused patients (52.9%) belonged to the American Society of Anesthesiologists (ASA) III category, but the need for blood transfusion did not differ significantly across ASA I, II, III, and IV categories (p=0.44) (Table 1). The grading of the ASA was completed by two qualified anesthetists according to the ASA classification [10].

**Table 1:** American Society of Anesthesiologists - Classification Grading Frequencies and Percentages * Percentage corrected to 1 decimal place ASA: American Society of Anesthesiologists; n: sample

ASA Grade	Frequency (n)	Percentage (%)*	Mean 81.2 Standard Deviation 104.3
1	14	2.8	
2	137	27.3	
3	263	52.5	
4	77	15.4	
5	7	27.3	
Missing	3	0.4	
Total	501		

The mean time to surgery was significantly longer in the transfused group (35.8 hours) compared to the non-transfused group (31.2 hours) (p=0.035). The average LOHS was significantly longer in the transfused group (22 days) compared to the non-transfused group (16.9 days) (p=0.022). The study population consisted of a considerable proportion of elderly patients, with 68% of the patients being in the higher subset of the elderly population. Additionally, a substantial proportion of the patients had a higher ASA grade, indicating a higher degree of co-morbidities. The ASA grade is a commonly used surrogate marker for assessing the preoperative co-morbidities index of patients. This indicates that the study cohort was representative of a population with significant preoperative risk factors that could potentially impact surgical outcomes (Table 2).

**Table 2:** ASA Grade Description in Relation to Age Brackets of eNOFF Patients - Frequencies and Percentages * Percentage corrected to zero decimal place ASA: American Society of Anesthesiologists; eNOFF: elderly neck of femur fractures

Age Bracket (years)	ASA Grade 1	%	ASA Grade 2	%	ASA Grade 3	%	ASA Grade 4	%	ASA Grade 5	%	Missing	%
30-39	-	-	-	-	1	100%	-	-	-	-	-	-
40-49	1	14%	2	29%	4	57%	-	-	-	-	-	-
50-59	1	8%	7	58%	2	17%	2	17%	-	-	-	-
60-69	4	9%	15	34%	21	48%	4	9%	-	-	-	-
70-79	1	1%	34	29%	61	52%	18	15%	2	2%	1	1%
80-89	5	2%	56	26%	121	56%	33	15%	1	-	1	-
90-99	2	2%	22	23%	51	53%	18	19%	4	4%	-	-
100-110	-	-	1	20%	2	40%	2	40%	-	-	-	-
Total	14	3%	137	27%	263	53%	77	15%	7	1%	2	-

Table 3 presents the distribution of blood transfusions among eNOFF patients based on their ASA grading. The ASA grading system is a widely used tool to assess the overall health status of patients before surgery, and it is based on a scale from I to V, with I indicating a healthy patient and V indicating a patient with severe systemic disease. The results show that a higher proportion of eNOFF patients with a higher ASA grade (ASA III and IV) received blood transfusions compared to those with a lower ASA grade (ASA I and II). This observation is in line with the known association between higher ASA grading and increased comorbidities, which may lead to a higher risk of bleeding and the need for transfusions during surgery. Therefore, these findings suggest that the ASA grading can be a useful predictor of transfusion requirements in eNOFF patients and may help healthcare providers in optimizing patient care and managing the risks associated with transfusions.

**Table 3:** Transfusions In Relation to their Age Brackets according to their ASA Grading Distribution ASA: American Society of Anesthesiologists

Age Bracket (years)	ASA Grade 1	ASA Grade 2	ASA Grade 3	ASA Grade 4	ASA Grade 5
30-39	-	-	-	-	-
40-49	-	-	2	-	-
50-59	-	2	-	-	-
60-69	-	3	1	-	-
70-79	1	3	14	2	2
80-89	1	5	24	7	1
90-99	-	4	6	3	4
100-110	-	-	1	-	-
Total	2	17	48	12	7

**Figure 1:**
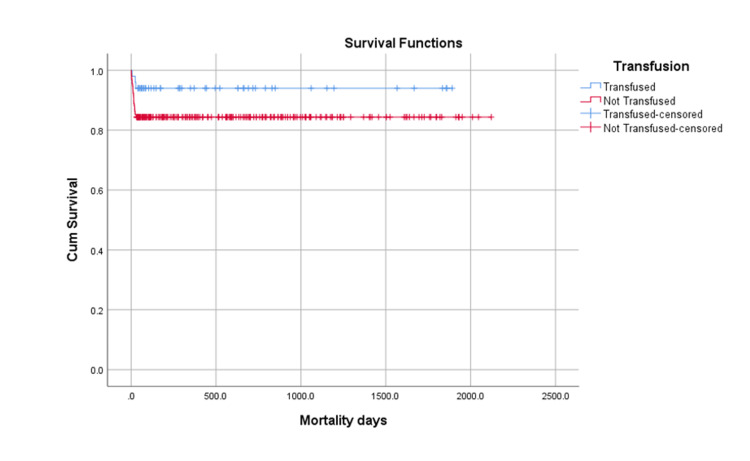
Graphical Representation of the Survival of Mortality at 30 Days Transfused group: 3 (4%);  Non-transfused group: 38 (9%) Not statistically significant difference;  Log-rank (Mantel-Cox) p-value: 0.07

Figure 1 shows that the mortality rate was lower in the transfused group (4%) compared to the non-transfused group (9%). However, the difference was not statistically significant (p=0.07).

**Figure 2:**
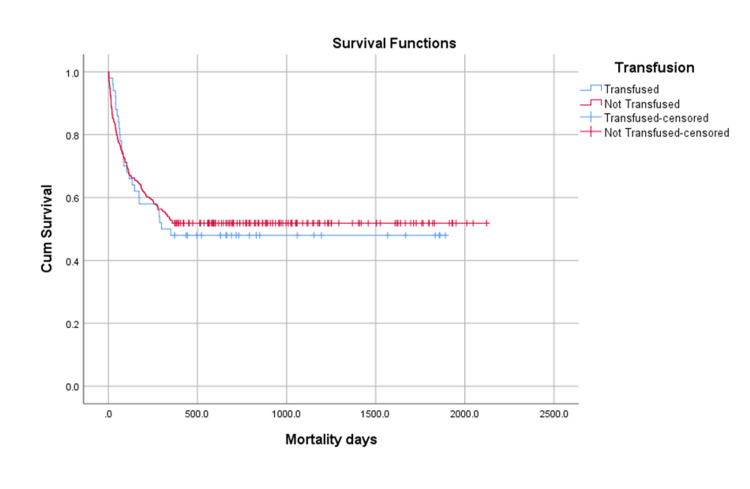
Graphical Representation of the Survival of Mortality at One Year Transfused group: 26 (33%);  Non-transfused group: 117 (28%) Not statistically significant difference; Log-rank ( Mantel-Cox) p-value: 0.789

Out of the total 501 patients, 293 (58.4%) died during the five-year post-operative follow-up period. The early post-operative 30-day mortality rate was lower in patients who received blood transfusion (4%) compared to those who did not receive blood transfusion (9%). However, this difference in mortality was not statistically significant (p=0.07). At one year post-surgery, mortality was higher in the transfused group (33%) compared to the non-transfused group (26%) (Figure 2).

**Figure 3:**
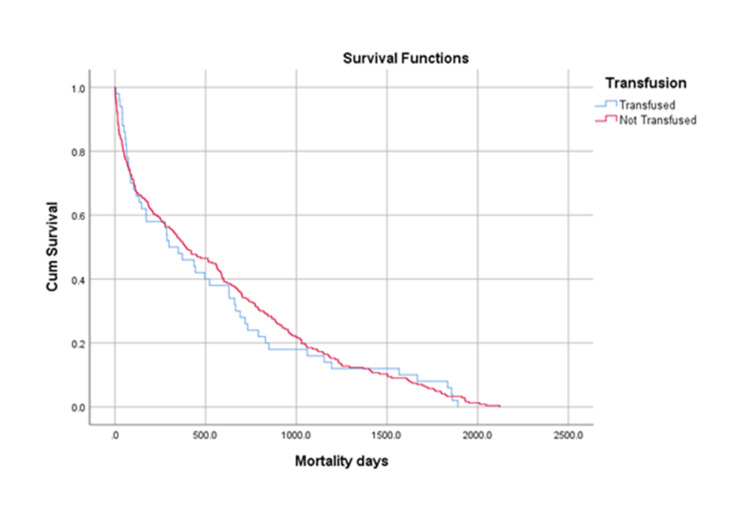
Graphical Representation of the Survival of Mortality at Five Years Transfused group: 50 (63.2%);  Non-transfused group: 243 (57.5%) Not statistically significant difference; Log-rank (Mantel-Cox) p-value: 0.7OO

Long-term five-year mortality rates remained higher in the transfused group (63.2%) compared to the non-transfused group (57.5%) (Figure 3). There was no statistical difference observed in the early post-operative (p=0.07), one-year (p=0.79), and long-term five-year mortality rates (p- 0.70) between patients in the transfused and non-transfused group.

## Discussion

The findings of this study support the practice of peri-operative blood transfusion in patients with low hemoglobin at presentation or in the early postoperative period as this reduces early postoperative mortality. The practice of blood transfusion does not cause any long-term deleterious effects or alterations in the physiology of the patient as the one-year and long-term five-year mortality rates are comparable for the eNOFF patients who did/did not receive a peri-operative blood transfusion. This positive effect on early postoperative mortality was also associated with statistically significant but marginally increased time to surgery and moderately increased post-operative LOHS.

The population that suffers from eNOFF is usually medically compromised and suffers from one or more medical co-morbidities. These patients also tend to have poor physiological reserves. The outcome of the management of these patients is very dependent on the medical co-morbidities that they suffer from. This also influences healthcare costs because of increased medical support and delayed rehabilitation [1].

One of the common peri-operative medical conditions in eNOFF patients is anemia. This could be pre-existing chronic nutritional anemia or because of blood loss from fracture or surgery. Anaemia in eNOFF patients influences the time to surgery, early postoperative recovery and rehabilitation, in-hospital stay, and healthcare costs [5]. If not optimized, these patients are also at higher risk of mortality, especially the ones with known cardiovascular co-morbidities. The use of blood transfusion for patient optimization is a common practice. The criteria used for transfusing patients can either be restrictive (hemoglobin (Hb) <9.7g/dL) or liberal (Hb <11.3 g/dL), which is mostly used as local trust policies in hospitals. In our series, we used a restrictive transfusion strategy often backed up with the clinical vital parameters and patients’ physiological state as well as symptoms. Earlier, it was the consensus to use the restrictive treatment strategy. Even the 2018 Frankfurt consensus on patient blood management recommends a threshold hemoglobin level for considering blood transfusion of <8g/dL for eNOFF patients in the peri-operative period. But the recently published evidence does not show any difference in the complications after liberal or restrictive transfusion strategy, but the liberal group does achieve better functional outcomes at one-year follow-up. This is shown in the results of the published work by Gregersen et al. [11-14].

The incidence of blood transfusion in eNOFF patients in our series was 15.8%. This was much less than the reported incidence of blood transfusion in a nationwide study from the Republic of Korea, which is about 74% [15]. Our study found that patients who required a blood transfusion had a significantly longer time to surgery and overall LOHS compared to those who did not. These findings are consistent with the results of previously published studies [5,16]. In this study, we did not evaluate the reasons for the delay in surgery and increased LOHS. Other potential reasons that can contribute to these include but are not limited to late presentation, other medical comorbidities, cancellation for lack of theatre capacity, lack of home support, and delays in arranging community care or rehabilitation/nursing home placement.

Mortality in eNOFF patients is a well-studied outcome. Though the figures vary across studies, the national 30-day mortality following eNOFF, as reported in the NHFD 2021 report is 8.3% [1]. This further increases to about 30% at the one-year follow-up [1]. Blood transfusion given to correct anemia can influence the mortality rate following the management of eNOFF patients. The increased blood volume from blood transfusion can contribute to increased cardiac workload and in turn contribute to cardiovascular complications in the already compromised physiology of the eNOFF patients [6,17]. It can also alter the electrolyte balance resulting in hyperkalemia and metabolic acidosis [7,8]. The physiological and metabolic alterations secondary to blood transfusion can influence mortality in eNOFF patients. The published literature is divided on the effect of blood transfusion on mortality in eNOFF patients. In some studies, it is a recognized risk factor for mortality while other studies fail to identify any direct contribution of blood transfusion to the mortality rates [15,18,19]. In our study, we evaluated the 30-day, one-year, and five-year mortality in eNOFF patients who received a blood transfusion and compared the findings to the group who did not.

Blood transfusion is beneficial for the eNOFF patients when it is clinically indicated and, in the early postoperative period, the one-month mortality is less than the no blood transfusion cohort. Though this difference is not statistically significant, there is a clear trend of the benefit of blood transfusion as most of the patients who needed blood transfusion belonged to the high-risk group and belonged to the ASA III category. The long-term one-year and five-year mortalities were comparable in eNOFF patients who did or did not receive a blood transfusion. Thus, blood transfusion does not cause any deleterious long-term effects on eNOFF patients.

Limitations

Our study had several limitations, including its retrospective design conducted at a single hospital, which may limit the generalizability of our findings. Furthermore, we did not conduct detailed analyses of individual co-morbidities in eNOFF patients who required blood transfusions. While receiving a blood transfusion was found to be associated with a longer time to surgery and LOHS, our study groups were not propensity-matched and statistical significance was not adjusted for observed differences. Therefore, it is important to exercise caution when attributing these outcomes solely to blood transfusion. Further research is needed to better evaluate the potential impact of confounding factors on these outcomes.

## Conclusions

Blood transfusion in eNOFF patients is a contentious issue. While it may be beneficial in reducing acute peri-operative mortality rates in high-risk patients, its impact on long-term mortality rates is inconclusive. Administering blood transfusion to eNOFF patients should be based on careful clinical assessment of the potential risks and benefits. Although blood transfusion can stabilize Hb levels and improve physiological reserves in high-risk patients, it also carries potential complications, including transfusion reactions and infections.

Blood transfusion should not be considered a definitive solution for improving long-term outcomes in eNOFF patients, and its use should be carefully balanced against potential risks and benefits, with close monitoring and follow-up of patients being crucial for optimizing clinical outcomes.
